# Tepotinib‐Induced Cholangitis in a Patient With Non‐Small Cell Lung Cancer: A Case Report

**DOI:** 10.1002/rcr2.70406

**Published:** 2025-11-11

**Authors:** Yoshimasa Hachisu, Kojiro Yoshida, Yuki Hosino, Kenta Ito, Shogo Uno, Kazuma Ezawa, Hirotaka Arai, Takeo Horie

**Affiliations:** ^1^ Department of Respiratory Medicine Maebashi Red Cross Hospital Maebashi Japan; ^2^ Department of Gastroenterology Maebashi Red Cross Hospital Maebashi Japan

**Keywords:** drug induced cholangitis, hepatotoxicity, lung cancer, MET‐TKI, tepotinib

## Abstract

Tepotinib, a mesenchymal epithelial transition factor (MET) tyrosine kinase inhibitor, is used to treat non‐small cell lung cancer with MET exon 14 skipping mutations. Although hepatotoxicity has been reported, drug‐induced cholangitis has not been reported before. Here, we report a case of tepotinib‐induced cholangitis in a 77‐year‐old woman with preexisting primary biliary cholangitis. During tepotinib treatment, the patient experienced abdominal pain along with elevated hepatobiliary enzyme levels. Imaging and histological examinations revealed findings similar to sclerosing cholangitis. The liver enzyme levels decreased after drug discontinuation. This case highlights the need for careful monitoring of hepatobiliary function when prescribing MET inhibitors, particularly in patients with underlying liver disease.

## Graphical abstract

Here, we report the first case of tepotinib‐induced cholangitis. When using MET inhibitors for chemotherapy, attention should be paid not only to treatment effectiveness but also to possible hepatobiliary side effects.

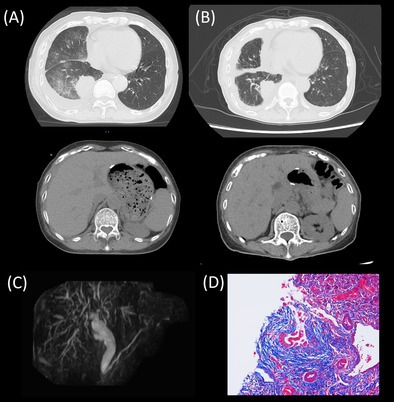

## 1 Introduction

In recent years, numerous tyrosine kinase inhibitors have been developed, leading to improved overall survival in patients with various malignancies. Their adverse effects are also recognised and may necessitate treatment discontinuation depending on the severity.

Tepotinib is a tyrosine kinase receptor inhibitor effective against non‐small cell lung cancer with mesenchymal epithelial transition factor (MET) exon 14 skipping mutations. Several adverse effects are known. Although tepotinib‐associated liver injury has been reported, drug‐induced cholangitis has not been previously reported. We encountered a case of drug‐induced cholangitis with tepotinib and report this case with a literature review.

## 2 Case Report

**FIGURE 1:**
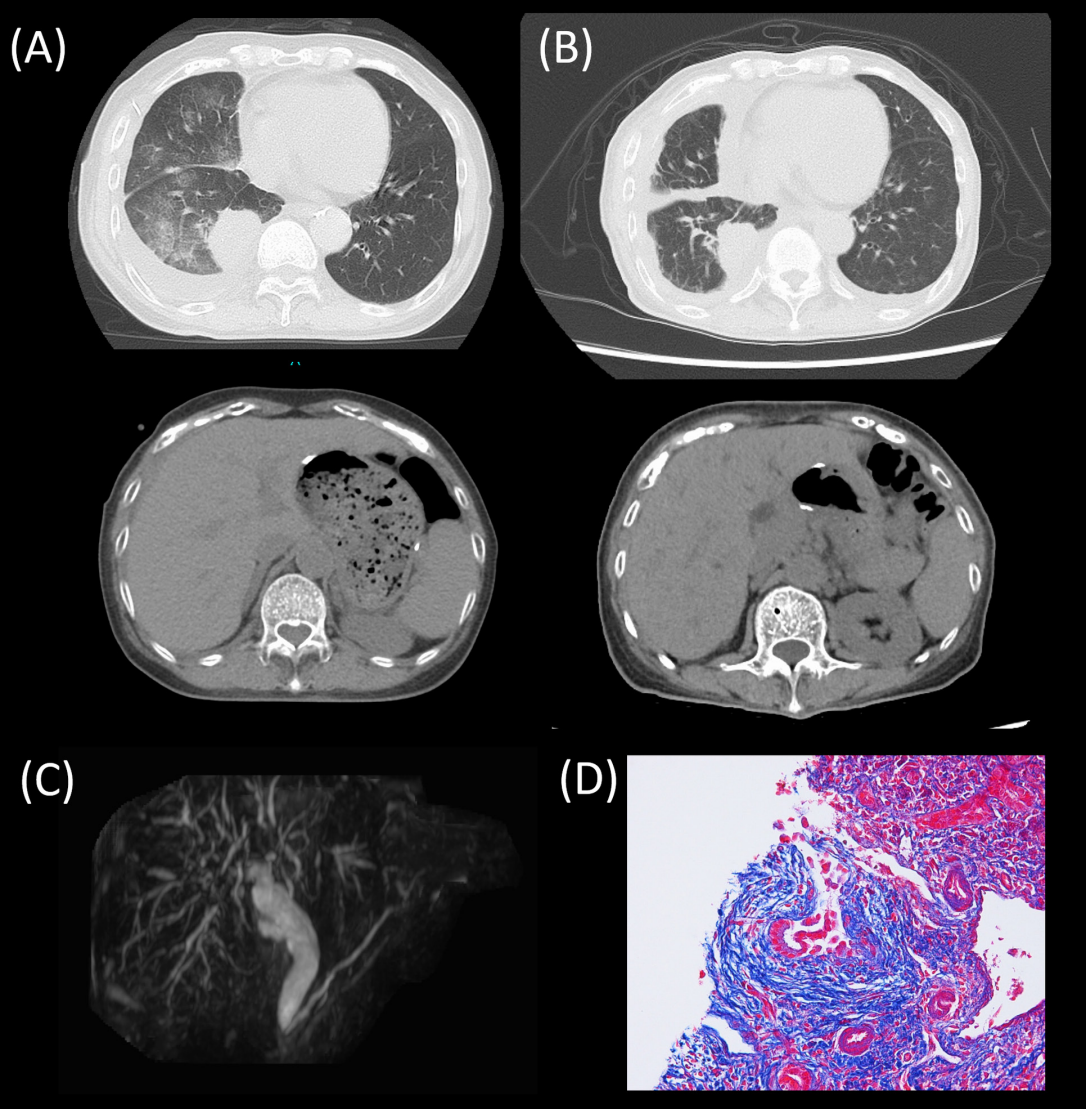
Imaging progression of lung cancer and cholangitis. (A) Computed tomography (CT) at the time of lung cancer diagnosis shows a mass in the right lower lobe and right pleural effusion. No obvious intrahepatic bile duct dilatation is identified. (B) CT taken at the time of elevated liver enzymes showed a slight reduction in the right lower lobe mass but revealed intrahepatic bile duct dilatation. (C) MRCP revealed a mixture of intrahepatic and extrahepatic bile duct strictures and dilatations. No obvious origin of obstruction was identified. (D) In the liver biopsy specimen, partial transmural fibrosis is observed around the bile ducts (Azan staining, X400).

A 77‐year‐old woman with a history of primary biliary cholangitis (PBC) was treated with ursodeoxycholic acid at another hospital. There were no findings suggestive of sclerosing cholangitis. In January, she visited another clinic with cough and dyspnea. Chest radiography revealed a right‐sided pleural effusion, computed tomography (CT) revealed a mass in the right lower lobe (Figure 1A). She visited our hospital on February 5, and no abdominal symptoms were observed. The Child‐Pugh score was 6 points, and no elevation of liver enzymes was observed (Table 1). A transbronchial lung biopsy was performed, confirming adenocarcinoma. Multiplex genetic testing identified a MET exon 14 skipping mutation and PD‐L1 expression of 1%. To manage pleural effusion, pleurodesis was performed on March 3, followed by the initiation of tepotinib 500 mg/day on March 6.

**TABLE 1:** Haematological data at first visit.

White blood cells	10,100/μL	Total protein	6.7 g/dL	KL‐6	119 U/mL	
Neutrophils	75.0%	Albumin	3.2 g/dL	BNP	26.9 pg/mL	
Lymphocytes	14.7%	Total bilirubin	0.3 mg/dL	Anti‐nuclear antibody	40 titer	
Eosinophils	3.1%	AST	17 U/L	CEA	53.9 ng/mL	
Monocytes	6.7%	ALT	9 U/L	CA19‐9	13.0 U/mL	
Basophils	0.5%	ALP	153 U/L	CYFRA	12.1 ng/mL	
Haemoglobin	14.2 g/dL	γ‐GTP	12 U/L	ProGRP	44.6 pg/mL	
Haematocrit	45.3%	LDH	192 U/L			
Platelets	32.2/μL	Bun	17 mg/dL	HBs‐antigen	Negative	
		Creatinine	0.91 mg/dL	HCV‐antibody	Negative	
PT‐INR	0.99	Sodium	141 mEq/L	IgG	2243 mg/dL	
PT%	100%	Potassium	3.7 mEq/L	IgG4	50.0 mg/dL	
APTT	27.0 s	Chloride	106 mEq/L	Anti‐mitochondrial antibody	428 U/mL	
Fibrinogen	553 mg/dL	Calcium	8.9 mg/dL			
D dimer	1.4 μg/mL	CK	44 U/L			
		C‐reactive protein	0.22 mg/dL			
		Glucose	138 mg/dL			
		HbA1c	5.9%			

**FIGURE 2:**
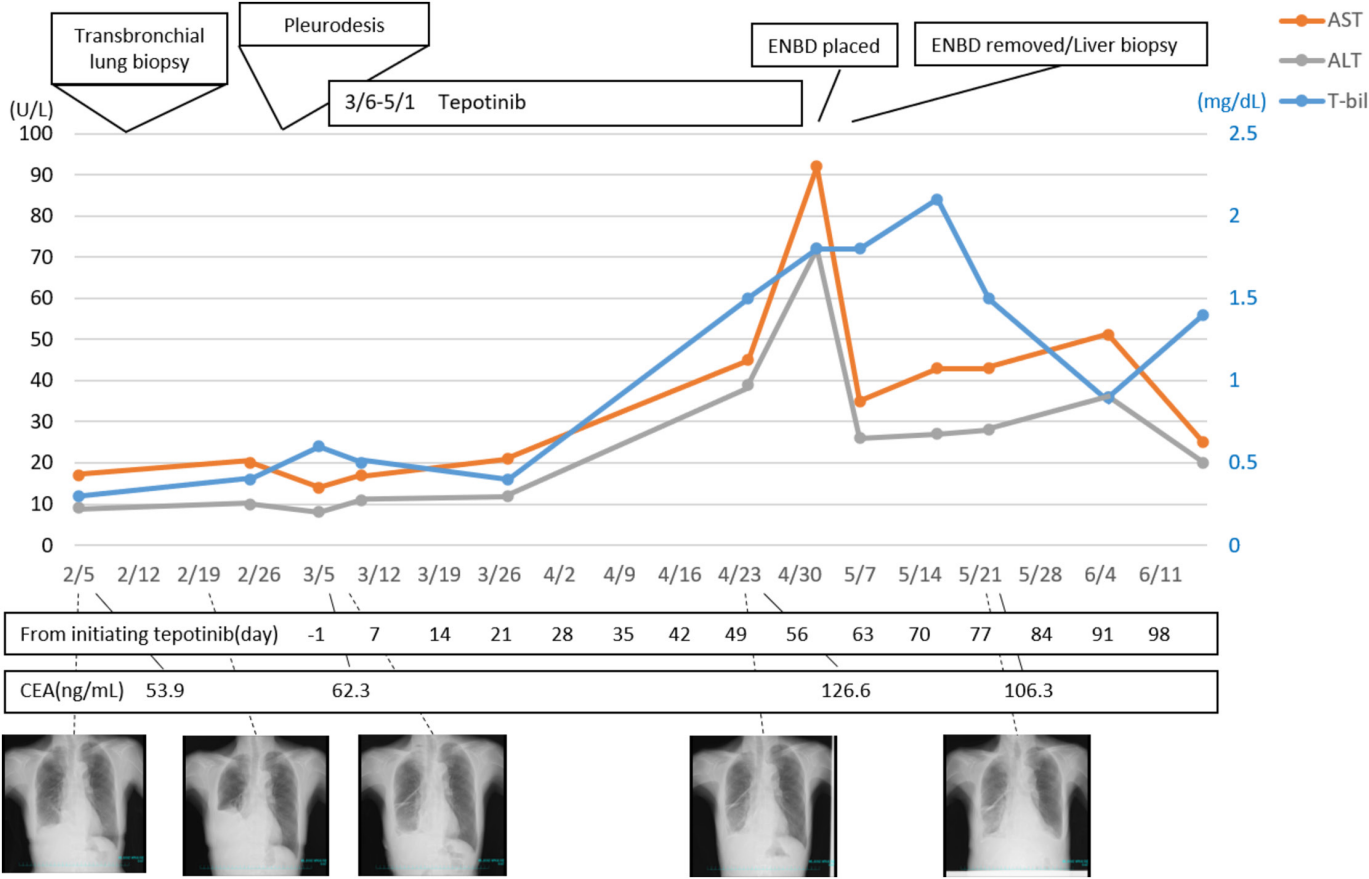
Treatment course and changes in liver enzymes, total bilirubin, CEA, and Chest x‐ray. Red and grey lines indicate changes in AST and ALT levels, respectively. The blue line indicates the change in total bilirubin level.

On April 24 (Day 50 after initiating tepotinib), the patient developed abdominal pain with elevated liver enzyme and inflammatory marker levels (Figure 2). CT revealed the regression of the right lower lobe mass and dilatation of the intrahepatic bile duct (Figure 1B). MRCP revealed a combination of intrahepatic and extrahepatic bile duct strictures and dilatations, suggesting sclerosing cholangitis (Figure 1C). No obvious source of obstruction was identified. Empirical oral amoxicillin‐clavulanic acid was administered for 7 days to treat a possible infection, but the symptoms did not improve.

On May 2 (day 58), laboratory tests demonstrated further elevation of the liver and biliary enzyme levels. Tepotinib was discontinued the same day due to suspected drug‐induced cholangitis. Endoscopic retrograde cholangiopancreatography revealed no obstructive bile duct lesions. Although an endoscopic nasobiliary drainage (ENBD) tube was placed, liver enzymes levels improved only minimally, effectively ruling out obstruction‐related cholangitis. On May 7 (Day 63), the ENBD tube was replaced, and a liver biopsy was performed. This revealed fibrosis around the bile ducts similar to sclerosing cholangitis (Figure 1D). Because there were no findings suggestive of primary sclerosing cholangitis on a prior liver biopsy, and abdominal symptoms with elevated liver and biliary enzymes after initiation of tepotinib, she was diagnosed with tepotinib‐induced cholangitis. After stopping tepotinib, her liver enzyme levels gradually decreased.

## 3 Discussion

Tepotinib is a MET tyrosine kinase receptor inhibitor used to treat non‐small cell lung cancer harbouring MET exon 14 skipping mutations. Reported adverse events include edema, nausea, abdominal pain, and hepatotoxicity, with elevated liver enzymes occurring in 5.9%–8.6% of cases [1]. However, the mechanism underlying hepatotoxicity remains unclear. For capmatinib, another MET inhibitor, it has been suggested that c‐MET inhibition may promote hepatocyte apoptosis [2]. However, to our knowledge, drug‐induced cholangitis caused by MET inhibitors has not yet been reported.

In a study on capmatinib, portal fibrosis was observed on liver biopsy in a patient with liver dysfunction following immune checkpoint inhibitor (ICI) therapy [3]. The authors concluded that ICI administration, rather than MET inhibitors, was more likely to be responsible for liver injury, leaving the association between MET inhibitors and cholangitis uncertain.

In the present case, a patient with pre‐existing PBC developed cholangitis after the initiation of tepotinib therapy, with histological findings resembling sclerosing cholangitis. Although no cases of tepotinib‐induced cholangitis have been reported in clinical trials, intrahepatic cholangitis has been observed in dogs in repeated‐dose toxicity studies [4].

The mechanism underlying drug‐induced cholangitis in this case remains unclear. One possible explanation, based on animal data, is direct injury to the cholangiocyte epithelium by the drug. In addition, the patient's history of PBC suggests that autoimmune mechanisms may have contributed to it. Although liver function was relatively preserved in this case, close monitoring of abdominal symptoms and liver enzyme levels is essential when administering MET inhibitors to patients with underlying liver diseases.

A limitation of this study lies in the lack of diagnostic accuracy of drug‐induced cholangitis. Although uncommon, periductal fibrosis may occur as part of the progression of PBC, and the possibility that the histological findings in this case were attributable to PBC cannot be completely excluded.

## Author Contributions

Yo.H. managed the patients, collected the data, and wrote the original manuscript. All authors revised, reviewed, and approved the final version of the manuscript.

## Consent

The authors declare that written informed consent was obtained for the publication of this manuscript and the accompanying images using the consent form provided by the journal.

## Conflicts of Interest

The authors declare no conflicts of interest.

## Data Availability

The data that support the findings of this study are available from the corresponding author upon reasonable request.
